# Automated Extraction of Information From Texts of Scientific Publications: Insights Into HIV Treatment Strategies

**DOI:** 10.3389/fgene.2020.618862

**Published:** 2020-12-22

**Authors:** Nadezhda Biziukova, Olga Tarasova, Sergey Ivanov, Vladimir Poroikov

**Affiliations:** ^1^Laboratory of Structure-Function Based Drug Design, Department of Bioinformatics, Institute of Biomedical Chemistry, Moscow, Russia; ^2^Department of Bioinformatics, Faculty of Biomedicine, Pirogov Russian National Research Medical University, Moscow, Russia

**Keywords:** text mining, data mining, named entity recognition, NER, virus-host interactions, HIV, viremic control

## Abstract

Text analysis can help to identify named entities (NEs) of small molecules, proteins, and genes. Such data are very important for the analysis of molecular mechanisms of disease progression and development of new strategies for the treatment of various diseases and pathological conditions. The texts of publications represent a primary source of information, which is especially important to collect the data of the highest quality due to the immediate obtaining information, in comparison with databases. In our study, we aimed at the development and testing of an approach to the named entity recognition in the abstracts of publications. More specifically, we have developed and tested an algorithm based on the conditional random fields, which provides recognition of NEs of (i) genes and proteins and (ii) chemicals. Careful selection of abstracts strictly related to the subject of interest leads to the possibility of extracting the NEs strongly associated with the subject. To test the applicability of our approach, we have applied it for the extraction of (i) potential HIV inhibitors and (ii) a set of proteins and genes potentially responsible for viremic control in HIV-positive patients. The computational experiments performed provide the estimations of evaluating the accuracy of recognition of chemical NEs and proteins (genes). The precision of the chemical NEs recognition is over 0.91; recall is 0.86, and the F1-score (harmonic mean of precision and recall) is 0.89; the precision of recognition of proteins and genes names is over 0.86; recall is 0.83; while F1-score is above 0.85. Evaluation of the algorithm on two case studies related to HIV treatment confirms our suggestion about the possibility of extracting the NEs strongly relevant to (i) HIV inhibitors and (ii) a group of patients i.e., the group of HIV-positive individuals with an ability to maintain an undetectable HIV-1 viral load overtime in the absence of antiretroviral therapy. Analysis of the results obtained provides insights into the function of proteins that can be responsible for viremic control. Our study demonstrated the applicability of the developed approach for the extraction of useful data on HIV treatment.

## Introduction

Scientific publications represent the main source of knowledge for researchers in different fields of biology and medicine. Besides, the more pressing the problem for humanity is, the more articles devoted to this problem can be found in the repositories of scientific publications. The extraction of records from scientific publications provides the opportunity to analyze the information derived from primary sources; therefore, such an approach helps to obtain the most contemporary information (Cash, 2004; Tarasova et al., 2015, 2019; Saik et al., 2016). Currently, text-mining technologies aimed at rapid automated extraction of specific information are under rigorous development.

Analysis of interactions between named entities (NEs) representing proteins, genes, and chemical compounds can help investigate the particular molecular mechanisms of disease progression, the effect of drugs, and reveal the drug-drug interactions important for efficacy of therapy (Chen et al., 2014; Tannenbaum and Sheehan, 2014; Lim et al., 2016; Szklarczyk et al., 2019).

Identification of associations between NEs in the texts of scientific publications includes two steps: (i) extraction of named entities from the texts, and (ii) recognition of associations. This is the focus of the Named Entity Recognition (NER) methods. There are two main groups of approaches used for NER: (i) based on rules and dictionaries and (ii) based on machine learning methods. The main disadvantage of rule and dictionary-based algorithms is the inability to extract information about entities not included in dictionaries. Another drawback is the requirements for the allocation of memory for storing dictionaries.

Machine learning methods require sets of texts, in which the names of proteins, genes, chemical compounds, and so on are labeled by an expert or a group of experts. Then, using such texts as a training set, it is possible to adjust the algorithm to recognize NEs in a large number of articles. Finally, it is possible to identify relationships between the NEs extracted. Machine learning methods have advantages over dictionary-based methods because they provide the recognition of new NEs not included in dictionaries and, therefore, are the best option for the careful extraction of information. At present, many novel text corpora are constantly developing for the purposes of the scientific community and provide the possibility to extract information about a variety of NEs: genes, proteins and chemicals names, symptoms and syndromes of the diseases, side effects and toxicity of drugs, revealed during the clinical trials or as a result of medical studies, cases studies, etc.

There are methods aimed at NER that have been developing during the last years (Kaewphan et al., 2018; Korvigo et al., 2018; Hemati and Mehler, 2019; Hong and Lee, 2020; Huang et al., 2020; Kilicoglu et al., 2020). Most of them are based on algorithms for NER related either to chemicals or biological objects. In this study, we aim to develop and test an algorithm for the extraction of named entities of genes/proteins and chemical compounds and identify associations between them. Our method of NER is based on the conditional random fields and uses a set of originally developed word features that allow for context consideration. Thus, we suggest selecting a set of publications strictly relevant to the subject and extracting a set of chemical NEs, proteins, and genes to derive the NEs associated with one another. After that, their functions in a particular molecular mechanism of the disease can be analyzed.

Human immunodeficiency virus (HIV) still remains one of the challenges for humanity (Rojas-Celis et al., 2019; Tarasova et al., 2020). The number of new HIV cases per year reached 1.7 million (WHO, HIV incidence)^1^ while is, Number of People (All Ages) Living With HIV^2^ is around 38 million (WHO). Antiretroviral therapy (ART) helps to reduce viral load and disease progression, but antiretroviral medicine should be taken by a patient for term of life. The risk of HIV drug resistance and side effects of antiretroviral medicines decrease the effectiveness of ART (Iyidogan and Anderson, 2014; Tarasova and Poroikov, 2018). At the same time, an effective HIV vaccine does not exist (Ventura, 2020).

Taking into account the importance of the problem, we consider two main approaches for the HIV/AIDS treatment as case studies: (1) the usage of antiretroviral drugs and (2) studies of the ways of HIV/AIDS development in different groups of patients and attempts to affect the key proteins of the pathways providing a long period of disease progression. The mechanisms identified can be used for the development of novel strategies of HIV treatment and vaccine development. We have validated and tested our algorithm on the tasks of identification of (i) chemicals that can be considered as HIV inhibitors and (ii) groups of proteins that may be important for the different velocity of HIV/AIDS. To reach these purposes, we used the abstracts of publications relevant to HIV/AIDS treatment for case studies of HIV inhibition and HIV viremic control. More specifically, the algorithm developed is aimed at extracting (a) the names of HIV reverse transcriptase (RT) inhibitors and (b) the protein (gene) names described in articles relevant to the studies of HIV elite controllers, i.e., the group of HIV-positive individuals with an ability to maintain an undetectable HIV-1 viral load overtime in the absence of antiretroviral therapy.

## Materials and Methods

The extraction of the NE names includes several stages. First, we collected text corpora and made their preprocessing. Second, we developed an algorithm of NER and its parameter optimization. Third, we carried out validation and testing of an algorithm and analysis of the information obtained.

To extract NEs, we used annotated text corpora and applied an algorithm based on conditional random fields (CRF). Text annotation in the corpus implies an indication of NE position inside the text.

### Text Corpora

We used the CHEMDNER^3^ (Krallinger et al., 2015) and ChemProt^4^ as annotated corpora. Both corpora consist of freely available abstracts of articles.

CHEMDNER consists of three sets (training, evaluation, and development sets) and includes 10,000 abstracts. It has been developed for the purpose of chemical NER (Krallinger et al., 2015). Annotations include the position of NE in the text and also the NE type: ABBREVIATION (as ATP), FAMILY (as steroid hormones), FORMULA [as S or C (sulfur or carbon)], IDENTIFIER (as GRN-529), MULTIPLE (as nucleoside tri- and di-phosphates), SYSTEMATIC (as sphingosine-1-phosphate), TRIVIAL (as progesterone).

ChemProt also consists of three sets—training, development, and test—and includes 2,482 abstracts in total. It also includes annotations of genes, proteins, and chemical compounds. ChemProt includes the following types of NEs: (i) GENE-Y is for proteins/genes that can be normalized or associated with a biological database identifier, (ii) GENE-N is for proteins/genes that cannot be normalized or associated with a biological database identifier and (iii) CHEMICAL is for chemical compounds^4^. The proteins and genes are not considered in ChemProt separately, but search in the databases containing data on genes and proteins can help identify protein names and names of genes.

### Text Preprocessing

Algorithms for entity recognition in texts require tokenization. It is the process of splitting the whole text into elementary text units. As a result of tokenization, the text is presented as a set of tokens. Words, symbols, numbers, can be used as tokens. Thus, we used this method for all symbols and spaces implemented in the “wordpunct_tokenize” function of the NLTK Python library.

Tokens can be divided into groups: those that belong and do not belong to NEs. If an NE originally consists of two words (for example, reverse transcriptase) or includes any symbols (for example, sphingosine-1-phosphate), then after tokenization it will be presented by a list or array of tokens rather than a simple string. We used the labeling system SOBIE (Rocktäschel et al., 2012; Batista-Navarro et al., 2015; Dai et al., 2015; Leaman et al., 2015) to indicate the position of the term associated with a particular token.

SOBIE is an abbreviation for tags: “S” (Single)—if a token belongs to NE and NE consists of one token, “O” (Out)—if a word doesn't belong to NE, “B” (Begin) is a label of the first word of composite NE if NE consists of two or more words, “E” (End)—is a label of the last word of composite NE, if NE consists of two or more words and I (Inside)—is a label of words belong to NE that are between “B” and “E” if NE consists of three or more words. The example of labeling text by SOBIE is presented in Supplementary Figure 1.

Tokenized corpora texts with a set of SOBIE labels were placed to the database managed by PostgreSQL DBMS. The schema of the database is provided in Supplementary Figure 2.

We compiled a features set for each word to train a model. For NER, it is essential to take into account the context, which means the words should follow one another in order without mixing. In our model, each word W is characterized by its features along with the features of one word before and after W. Each token was described with a triple set of features (Table 1) - the features of a particular token and the features of two tokens: one before and one after the considered one.

**Table 1:** The set of features used for CRF.

**Feature**	**Type**	**Meaning**
Word	String	Word string
Lower	String	Lowercase word string
Isupper	Boolean	If word is uppercase
Istitle	Boolean	If word's first symbol is uppercase
Isdigit	Boolean	If word is digit
Hasdigits	Boolean	If word has digits
Isnonspecific	Boolean	If word belongs to non-specific terms list
Isstopword	Boolean	If word belongs to stop-words list
Hassymbols	Boolean	If word has symbols
Word[n-3:n]	String	Three last chars of word string
Word[n-2:n]	String	Two last chars of word string
Firstchar	String	Word's first char
Length	Integer	Number of chars in word string
Postag	String	Word's part of speech tag

*Features which do not belong to the standard set are highlighted by gray color*.

We have compiled a list of non-specific terms and used the belonging of a token to one of them as a feature. Non-specific terms are general words that can indicate the presence of the term in the proximity to NEs of a protein (gene) or a chemical compound in the text. For example, such terms may include the words “inhibit,” “chemical” for the names of chemical compounds and “target,” “genes” for the names of proteins and genes. The list of all these non-specific terms for NEs of proteins/genes and chemical compounds is presented in Supplementary Materials. We used marks “C” for non-specific terms used for chemical compounds and mark “G” – for proteins and genes. If a non-specific term was used in both lists, it received both labels: “C” and “G.”

**Figure 1:**
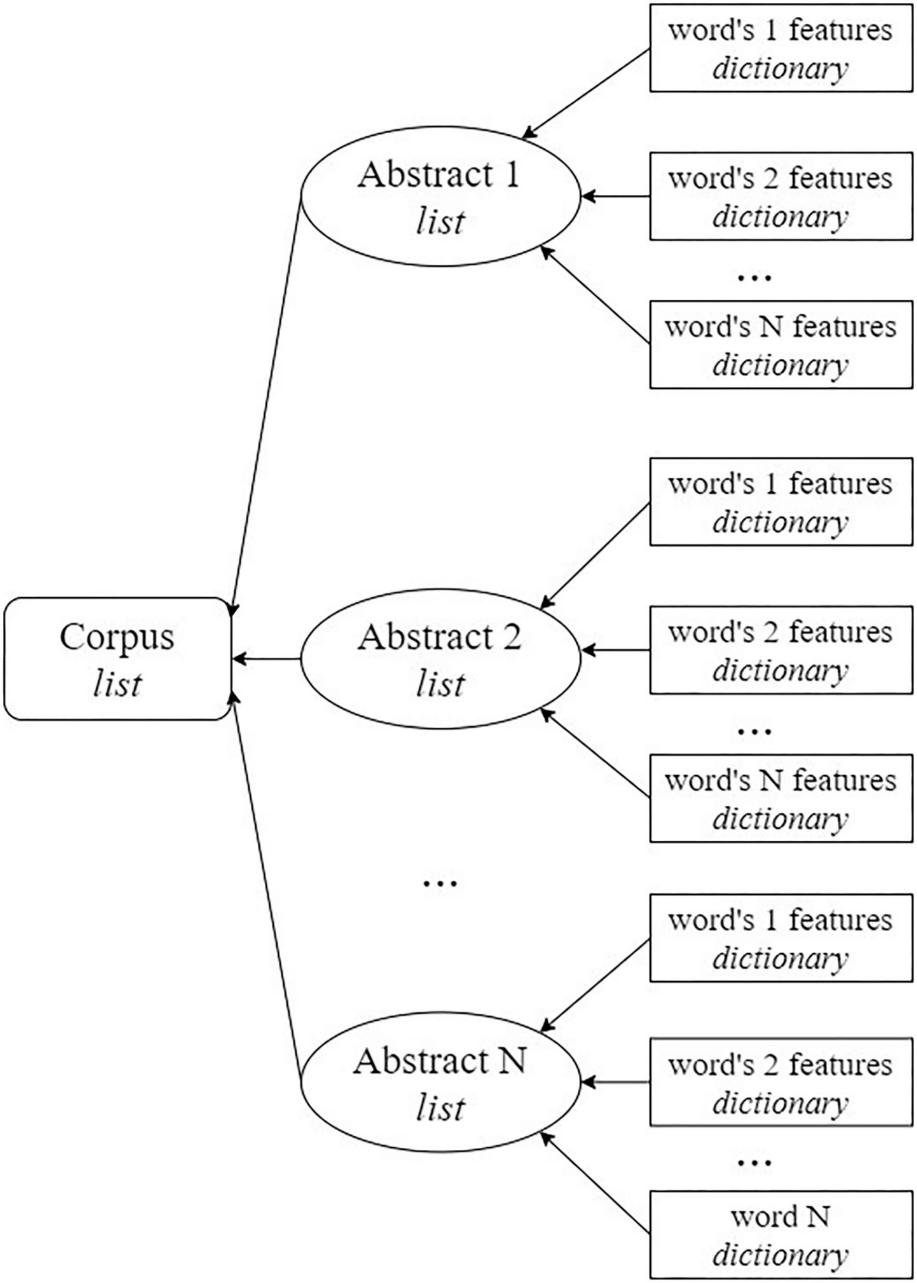
Scheme for the organization of data that are input for the models.

All the features were obtained using scripts prepared using Python 3.7. The features were represented as dictionaries to be able to access each feature individually by its keyword. Finally, the data were represented in the format that is shown in Figure 1.

### Algorithm Realization

As the next step, we built a model. For our approach, we used an algorithm named CRF in realization using Python 3.7 and SciKitLearn library. This library provides a lot of algorithms using machine learning, different metrics, etc. The CRF algorithm allows us to take the context of a phrase into account. We suggest that the set of features developed can help to improve the recognition of context near the NEs. We used the hyperparameter optimization function, which is included in the SciKitLearn to achieve the highest accuracy of the model.

We built several models. The first model (i) was built to provide the recognition labels of chemical entity mentions. The second model (ii) aimed at recognizing names of proteins and genes. The third model (iii) allowed recognition of types (ABBREVIATION, FORMULA, IDENTIFIER etc.) of chemical entity mentions. Based on the models (i–iii), we built the fourth model (iv) to extract chemical compounds and proteins/genes. This model combines algorithm for recognizing the names of chemical compounds and proteins and identifying the types of chemical compounds. Thus, the models (i) and (iii) were built based on CHEMDNER as a training corpus. The model (ii) was built using ChemProt. And the model (iv) was built using both CHEMDNER and ChemProt. Initially, models were tested using 5-fold cross-validation (Stone, 1974).

We used precision, recall, and F1-score to assess the quality of model recognition.

Precision is the proportion of positive identifications that was actually correct. It is calculated as the ratio of the number of true positive identification to the sum of true positive and false-positive identifications (1).

(1) $$\begin{array}{r} Precision=\;\frac{TP}{TP+FP}\;; \end{array}$$

Recall is the proportion of actual positive NE mentions that were identified correctly. It is calculated as the ratio of the number of true positive identifications to the sum of true positive and false negative decisions.

(2) $$\begin{array}{r} Recall=\;\frac{TP}{TP+FN}\;; \end{array}$$

F1-score is the harmonic mean of precision and recall.

(3) $$\begin{array}{r} F1=2\;\frac{precision\;•\;recall}{precision\;+\;recall}\;; \end{array}$$

The test set was formed based on the idea that if an article is strictly relevant to reverse transcriptase inhibition and our algorithm is able to recognize the NE of a protein and a name of a chemical compound or drug then there is a high probability that the chemical named entity identified is the name of HIV reverse transcriptase inhibitor.

We used the set of 148 publications abstracts collected from NCBI PubMed. We used the workflow developed earlier (Tarasova et al., 2019). In this workflow we were focused on the publications that included the description of HIV inhibitors and included the details of biological experiments used for their testing. Using the Python script, we automatically extracted over 15,000 abstracts of articles from PubMed using the query “HIV AND reverse transcriptase AND inhibitors.” Then, using the Lingpipe 4.1.2 tool (Carpenter, 2007), we selected a set of over 1,000 abstracts strictly relevant to the development, synthesis, and testing of HIV RT inhibitors. We used Lingpipe 4.1.2 since it provides the possibility of building the models of text classification into the classes according to the content, and the selection of the texts strictly relevant to the particular subject. Earlier we used Lingpipe 4.1.2 to perform selection of the abstracts of publications, which included the description of HIV-1 reverse transcriptase inhibitors including the details of their biological testing (Tarasova et al., 2019) with mean accuracy over 0.83. We assume that the data on the biological experiment details in the publication text can help to confirm its relevance to the inhibition of HIV-1 RT.

After that, we manually selected 148 of them, which consisted the description of HIV-1 reverse transcriptase inhibitors and the details of their biological testing. This set of abstracts was used to evaluate the accuracy of extracting the names of reverse transcriptase inhibitors.

The texts of abstracts can include the NEs of such chemical compounds as ATP, various ions, DNA, etc. If we want to extract the small drug-like compounds mainly, we would like to filter out ions and biological molecules remaining only drug-like compounds with molecular weight ranged from 300 to 700 (Da). So, to filter them out, we have additionally introduced filters for the classes of chemical compounds obtained based on the results of recognition using CRF. The classes FORMULA and FAMILY were excluded because the class FORMULA is mainly represented by ions, and the class FAMILY contains groups of chemical compounds that do not include the specific names of small molecule inhibitors of HIV RT.

Once we have developed an algorithm providing recognition of proteins (genes) names, we also tested the applicability of our algorithm to the extraction of proteins and genes responsible for the slow disease progressions of HIV-positive patients. To perform this analysis, we have collected a set of abstracts of publications from NCBI PubMed and NCBI PubMed Central (PMC) databases. We collected the abstracts strictly relevant to (1) the HIV elite controllers (ECs), a group of patients who do not progress into HIV/AIDS for years in the absence of antiretroviral therapy, and (2) the whole cohort of HIV-positive patients. The first set related to HIV elite controllers was obtained based on the query “(HIV[Title/Abstract] OR “human immunodeficiency virus”[Title/Abstract] OR “HIV”[Mesh] OR AIDS[Title/Abstract] OR “acquired immunodeficiency syndrome”[Title/Abstract] OR “Acquired Immunodeficiency Syndrome”[Mesh]) AND (“elite control^*^”[Title/Abstract] OR “Elite suppress^*^”*[Title/Abstract])*.” We obtained over 840 abstracts strictly relevant to the HIV elite controllers. The second set of abstracts was collected using the query “*HIV positive AND HIV/AIDS”* and included over 30 thousand of abstracts. We excluded from the group (1) the abstracts belonging to the group (2) because the group (2) may include HIV-positives ECs and we are interested in the differences between the protein profiles of groups (1, ECs) and (2, HIV-positives excluding ECs) mainly. The abstracts of groups (1) and (2) were processed using the NER algorithm developed, and names of proteins (genes) were extracted from them. Then, we excluded the proteins found in the abstracts of the group (2) from a list of proteins obtained for the group (1). We suggest that it provides the opportunity to compile a list of proteins responsible for the slow HIV/AIDS disease progression.

The results of our computational experiments are described and discussed below.

## Results

### Extraction of Chemical Named Entities

In our study, we performed several computational experiments using CHEMDNER and ChemProt corpora and the texts related to (i) the inhibition of HIV-1 reverse transcriptase and (ii) HIV ECs. Earlier (Tarasova et al., 2019), we collected a set of papers consisted mainly of those relevant to the development and testing of HIV reverse transcriptase (RT) inhibitors, the “HIV-RT-inhibitors corpus.” In the current study, we curated this corpus carefully and enlarged it with publications strictly relevant to the inhibition of HIV RT. The chemical compounds extracted were supposed to be inhibitors of reverse transcriptase based on a specific selection of texts for the test set described below.

We built the models for chemical and protein/gene NER based on CHEMDNER and ChemProt corpora, respectively, and calculated their accuracy using five-fold cross-validation. We performed several computational experiments for predicting SOBIE labels of belonging a token to a chemical and protein/gene named entity (NE) as well as prediction of certain types of chemical entity mention (ABBREVIATION, FORMULA, IDENTIFIER etc.). We evaluated the best way of NER using the features of text developed. The detailed description of text corpora and our computational experiments are provided in the section Materials and Methods.

First, we built a model to predict SOBIE—the labels for the parts of chemical NE: S - Single—for NE that contains one token; B, E—Begin, End—as the labels for the first and the last token of NE, respectively, if NE contains at least two tokens; I—Inside—as a label for the tokens that located between B and E, if NE includes three or more words; O—Out—as a label for the words that does not belong to NE. The results of five-fold cross-validation for annotated corpora CHEMDNER and ChemProt are represented in Table 2.

**Table 2:** Precision, recall, and F1-score for model that predicts SOBIE for chemical entity mentions.

	**Precision**	**Recall**	**F1-score**
S	0.906	0.8388	0.871
O	0.9908	0.996	0.9934
B	0.861	0.7898	0.8238
I	0.9216	0.8782	0.8994
E	0.8764	0.8032	0.8378
Avg	**0.9112**	**0.8612**	**0.8851**

*The average values of precision, recall and F1-score are given in bold*.

Table 2 displays that the recognition of chemical entity mentions occurs with reasonable accuracy (F1-score is 0.89). We carried out an experiment combining the CHEMDNER and ChemProt to recognize the names of chemical compounds. The volume of training set increased by more than two thousand articles (more than 25%). However, this did not lead to a significant increase in accuracy.

We assumed that the next task might require filtering the names of chemical compounds by their type. Thus, for example, the texts may contain the names of ions (Ca^2+^, Mn^2+^), which can be filtered out in case if we are focused on the small drug-like compounds only with molecular weight range from 300 to 700 (Da). Based on this conclusion, we built a model identifying the types of recognized names of chemical compounds. Accuracy was assessed with five-fold cross-validation and is presented in Table 3.

**Table 3:** Precision, recall, and F1-score for predicting types of chemical entity mentions.

	**Precision**	**Recall**	**F1-score**
ABBREVIATION	0.9092	0.8946	0.9016
FAMILY	0.8700	0.8074	0.8368
FORMULA	0.9176	0.9030	0.9098
IDENTIFIER	0.8574	0.8954	0.8748
MULTIPLE	0.8334	0.8288	0.8226
SYSTEMATIC	0.9008	0.9394	0.9196
TRIVIAL	0.9200	0.9198	0.9198
Avg	**0.8869**	**0.8841**	**0.8836**

*The types of chemical compounds are explained in Supplementary Material (Supplementary Table 1). The average values of precision, recall and F1-score are given in bold*.

From the values of precision, recall, and F1-score displayed in Table 3, one can conclude that filtering by the types of NEs of chemical compound can be used for the selection of the particular type of chemical NE of interest.

The algorithms obtained for recognition of the names of chemical compounds and their types and names of proteins (genes) were then combined and applied on a test set of texts devoted to inhibition of HIV reverse transcriptase. The prediction of genes and proteins names was obtained using the SOBIE labels only. The principle of the combined algorithm was the sequential recognition of the names of chemical compounds, their types, and then the names of proteins.

As a result, the names of proteins and chemical compounds were obtained. The rule for extraction of NEs of HIV RT inhibitors was the presence in the abstract of at least one recognized protein name and one named entity of chemical compound. So, in addition to the names of potential reverse transcriptase inhibitors, it is possible to extract NEs of chemical compounds that can interact with two HIV proteins, for instance, reverse transcriptase and protease.

Evaluation of the results was carried out so that if the recognized chemical compound is not an actual inhibitor of reverse transcriptase, then the number of false positives (FP) increased by one. If an inhibitor was encountered in the text and was not recognized, then the number of false negatives (FN) also increased by one.

Also, we evaluated the accuracy of the recognition of all chemical compounds in the text. The results are shown in Table 4.

**Table 4:** Precision, recall and F1-score obtained when testing the algorithm on a sample of texts devoted to the inhibition of HIV reverse transcriptase.

	**Precision**	**Recall**	**F1-score**
Chemical compounds	0.83	0.94	0.88
Reverse transcriptase inhibitors	0.80	0.97	0.88

Based on the value of the recall metric, we can conclude that, in general, we managed to extract almost all names of reverse transcriptase inhibitors and chemical compounds from the test set. However, there are wrong recognized inhibitors and chemical compounds too. This is in agreement with the overall value of accuracy of NEs recognition in the text according to the results of 5-fold cross-validation. As we have mentioned earlier, we applied filters on classes of chemical compounds to reduce the number of false-positive results. This step allowed us to increase the precision of value to 0.85. We built automatic queries to PubChem database^5^. For all recognized chemical named entities we obtained PubChem identifiers, if PubChem identifier was found.

We also tried to extract the names of chemical compounds from a set of texts dedicated to elite HIV/AIDS controllers. We assumed that if the texts contain proteins (genes) responsible for the non-progression of HIV/AIDS, then they can also indicate chemical compounds that slow down the progression of HIV/AIDS by influencing the protein (gene). Thus, using model (i), we recognized the names of chemical compounds in texts related to the non-progression of HIV/AIDS and then manually checked the presence of chemical compounds that slow down the progression of HIV/AIDS. Unfortunately, there are few texts of the set in which such chemical compounds are mentioned. We provide some examples of extracted chemical compounds in the Discussion section.

### Extraction of the Protein and Gene Names

As the next step, we aimed at testing the algorithm for the extraction of protein and gene names from the texts. We built CRF using SOBIE labels similar to the algorithm for the extraction of chemical named entities. The results of protein (gene) recognition based on ChemProt are provided in Table 5.

**Table 5:** Precision, recall, and F1-score for model that predicts SOBIE for names of proteins/genes.

	**Precision**	**Recall**	**F1-score**
S	0.8616	0.828	0.8442
O	0.9732	0.983	0.978
B	0.8314	0.7764	0.803
I	0.8444	0.8078	0.8254
E	0.834	0.7792	0.806
Avg	**0.86892**	**0.83488**	**0.85132**

*The average values of precision, recall and F1-score are given in bold*.

Despite the fact that the recognition of the named entities of genes and proteins is carried out with a slightly lower accuracy than the recognition of the NEs of chemical compounds, the prediction accuracy still remains reasonable.

We tested the algorithm developed for evaluation a performance of extraction of proteins responsible for HIV/AIDS control and non-progression. To obtain the results, we applied the developed algorithm and the model (ii) to the set of papers, relevant to (1) ECs and (2) the whole cohort of HIV-positive patients retrieved from NCBI PubMed and NCBI PMC databases. The number of proteins and genes extracted from the texts of group (1) and group (2) abstracts is given in Table 6. The full list of proteins extracted for each of the groups represented in Table 6 is given in the Supplementary Materials.

**Table 6:** Numbers of proteins (genes) names associated with different velocity of HIV/AIDS progression.

	**The number of proteins (genes) unique names retrieved**
ECs (group 1 of papers abstracts)	478
HIV-positive (group 2 of papers abstracts)	1,443
Overlap between groups 1 and 2	75
Proteins specific for group 1 only	403
Proteins specific for group 2 only	1,368

As Table 6 displays, there are different protein profiles of the names of genes and proteins extracted from the set of abstracts relevant to the ECs (group 1) and the overall group of HIV-positive patients. For further analysis, we automatically obtained the synonyms of protein (gene) names and UniProt^6^ identifiers for each name of gene or protein extracted from group 1 of abstracts (i.e., articles relevant to ECs). We also identified the main functions of the proteins of this group. The interpretation of our results is given below in the Discussion section.

## Discussion

### Comparison of the NER Algorithm With Earlier Developed Approaches

We have compared the models obtained for NER of chemical compounds and proteins/genes with those developed earlier by other authors. Earlier NER approaches reached on average F1-score between 77.70 and 88.06% before post-processing (Campos et al., 2015; Khabsa and Giles, 2015; Xu et al., 2015; Korvigo et al., 2018), and for recognizing proteins/genes. Taking into account the results provided, one can conclude that the accuracy of NER for our method is comparable with that of some methods developed earlier. We suggested modifying the text features that lead to an increase in the recognition accuracy: in particular, we expanded the list of non-specific terms used for the recognition of genes and proteins.

We tried to merge two corpora, CHEMDNER and ChemProt, to improve the accuracy of chemical compounds recognition. Despite the assumption that if we increase the number of examples to train the model, the accuracy may increase, this did not happen in our case. A detailed comparison of the precision, recall, and F1-score values for the recognition of chemical NE (i) based on CHEMDNER and (ii) merged CHEMDNER and ChemProt corpora and (ii) recognition of proteins and genes NE based on ChemProt corpus is provided in Supplementary Figure 1.

The lower recognition accuracy of chemical compounds based on the combined corpus may be due to ChemProt was primarily aimed at finding relationships between proteins and genes. Therefore, chemical NEs in ChemProt are less common in the texts compared to CHEMDNER.

The algorithms obtained for recognizing the names of chemical compounds and proteins (genes) were examined on the test sets of abstracts related to (i) the inhibition of HIV reverse transcriptase (ii) the identification of proteins associated with HIV control and non-progression.

### Extraction of Chemicals Names That Can Be Considered as Potential Medicines for HIV Treatment: A Case Study for HIV Reverse Transcriptase Inhibitors

From the texts relevant to the inhibition of HIV reverse transcriptase, we were able to extract inhibitors that actually exist and are currently used for the therapy. This allows us to conclude that using our method we can extract the names of chemical compounds considered as new inhibitors and can be used to treat HIV infection. The examples of the names of HIV reverse transcriptase inhibitors extracted from the texts of abstracts with their PubChem identifiers and their chemical structures are shown in Supplementary Figure 4.

### Identification of the Chemical Compounds Responsible for the Velocity of HIV/AIDS Progression: A Case Study for HIV Elite Controllers

Many of extracted compounds from elite controllers test set were parts of HAART or names of amino acids. But among all extracted compounds we were able to detect some that influenced HIV/AIDS progression.

For example, the article by Bermejo et al. (2016) describes the effect of the tyrosine kinase inhibitor dasatinib. During T-cell activation phosphorylation of SAMHD1 allows HIV infection. Dasatinib stopped SAMHD1 phosphorylation, which led to disruption of HIV reverse transcription.

Joshi et al. (2016) reported the relationship between heat shock protein 90 (Hsp90) inhibitors and HIV transcription. It has been shown that administration of Hsp90 inhibitors tanespimycin [17- (allylamino)−17-demethoxygeldanamycin] and AUY922 durably prevented viral rebound in mice.

As was mentioned above (in Results section) there were not plenty recognized chemical compounds that may lead to HIV/AIDS non-progression. But some of them were extracted, it demonstrates the usefulness of the approach developed and the possibility of working out this direction in the future.

### Identification of the Proteins and Genes Responsible for the Velocity of HIV/AIDS Progression: Case Study for HIV Elite Controllers

Once we were able to extract the set of protein and gene names from the texts relevant to HIV/AIDS ECs, we aimed to automatically identify the main biological processes and functions associated with them based on Gene Ontology (Gene Ontology Consortium, 2015) terms available from UniProt.

Automated queries to the UniProt database allow us to identify the belonging of a protein or a gene to either organism “homo sapiens” or a virus. There were some NEs associated with HIV, such as “gag-pol protein” or “pol peptide.” Also, we found that 11 names were not associated with any proteins, they represent false-positive results of our algorithm. Therefore, the automated verification using a database or a dictionary of proteins can help filter out the named entities that represent false positive results and therefore improve the recognition accuracy obtained using CRF (Song et al., 2015; Perera et al., 2020). It also helps select the names of protein belonging to the species of interest. We selected only proteins that were found in UniProt database and included the names of a protein extracted as one of the synonyms of names presented in UniProt. Therefore, some of proteins were filtered out because they were not associated with one unique record in UniProt.

As a result of automated processing of the files with gene/protein identifiers and their GO terms, we collected the most important biological processes associated with proteins extracted. They can be can be associated with HIV infection and can have an impact on the velocity of HIV/AIDS disease progression. We divided them into several groups according to the function most important for HIV-progression (see Table 7).

**Table 7:** The main functions of some proteins and genes found in the Uniprot database by the search using named entities extracted from abstracts of publications selected by their relevance to the description of HIV elite controllers.

	**The list of proteins**
Immune response	Human leukocyte elastase; nkg2a; nkg2d receptor; nkp30; nkp44; antileukoproteinase (ALP), apolipoprotein A-I, c4b-binding protein alpha chain (C4bp), complement C4-B (Basic complement C4), interferon-gamma; Calcitonin gene-related peptide 1 (Alpha-type CGRP); Antileukoproteinase (ALP); C4b-binding protein alpha chain (C4bp); Complement C4-B (Basic complement C4); HLA class I histocompatibility antigen (HLA-B27K protein) (MHC class I antigen) (Major histocompatibility complex); HLA-B alpha chain (B*5703GB) (MHC class I antigen); Interferon beta (IFN-beta) (Fibroblast interferon), Apolipoprotein A-I (Apo-AI); Interferon beta (IFN-beta) (Fibroblast interferon)
Autophagy	Alpha-1A adrenergic receptor; putative peripheral benzodiazepine receptor-related protein; forkhead box protein O3; interferon gamma; micotubule-associated proteins 1A/1B; microtubule-associated protein 1S (MAP-1S); microtubule-associated protein tau (Neurofibrillary tangle protein); Platelet-activating factor acetylhydrolase IB subunit beta; Microtubule-associated proteins 1A; Nicotinamide phosphoribosyltransferase (NAmPRTase)
inflammatory response	Adenosine deaminase (EC 3.5.4.4) (Adenosine aminohydrolase); angiotensin-converting enzyme 2; antithrombin-III (ATIII) (Serpin C1); calcitonin; 5'-nucleotidase (5'-NT); Integrin beta-2; human leukocyte antigen b57; interferon-gamma; tumor necrosis factor(nf)- alpha, interleukin (il)-2, C-reactive protein; Interferon gamma (IFN-gamma) (Immune interferon); Platelet factor 4 (PF-4); prostaglandin F2-alpha receptor (PGF receptor) (PGF2-alpha receptor) (Prostanoid FP receptor); prothrombin (EC 3.4.21.5) (Coagulation factor II)
Negative regulation of T cell mediated cytotoxicity	Carcinoembryonic antigen-related cell adhesion molecule 1; leukocyte immunoglobulin-like receptor subfamily B member 1 (CD85 antigen-like family member J); leukocyte immunoglobulin-like receptor subfamily B member 1 (LIR-1); HLA class I histocompatibility antigen, alpha chain G (HLA G antigen)
Negative regulation of CD8-positive, alpha-beta T cell activation	Leukocyte immunoglobulin-like receptor subfamily B member 1 (LIR-1) (cd85j receptor)
Regulation of gene expression	Progonadoliberin-1 (Progonadoliberin I), lhrh, Prostaglandin F2-alpha receptor (PGF receptor); Angiotensin-converting enzyme (ACE); Myc proto-oncogene protein (Proto-oncogene c-Myc); Calcitonin receptor (CT-R); Leukocyte immunoglobulin-like receptor subfamily B member 1 (LIR-1); Core histone macro-H2A.2, C-reactive protein; Estrogen receptor (ER); Pro-epidermal growth factor (EGF); Fibronectin (FN); Interferon gamma (IFN-gamma)

For some proteins, the names of which had been extracted from the texts of publications relevant to the studies of HIV ECs, we found direct associations of these proteins with the HIV progression (Taylor et al., 2000; Oleksyk et al., 2009; Marras et al., 2013; Slavov et al., 2015; Roy et al., 2017; Parodi et al., 2019; Hersberger et al., 2020; Wendel et al., 2020). We provide a few examples of the association between biological processes known for the proteins identified and their possible role in HIV disease progression. For instance, H. Fausther-Bovendo and co-authors reported that the increased expression of NKp44L was observed in CD4+ T cells of HIV-positive patients (Fausther-Bovendo et al., 2009); that leads in an increased sensitivity of NKp44LCD4 T cells to the NK lysis activity. The cd85j receptor (LIR-1) was extracted from abstracts and is found to be associated with negative regulation of CD8-positive T cell activation according to UniProt data (Table 7). Also, there are data on its role in the control of HIV-1 replication in autologous dendritic cells (Scott-Algara et al., 2008). Also, we found the studies aimed at the identification of the interactions between S100A9 protein (a calcium-binding protein of the S100 family) and cd85j receptor. In particular, it was shown that HIV-1 infection modulates S100A9 expression on the surface of the monocyte-derived dendritic cells. Interaction between S100A9 protein and cd85j receptor, in turn, can have an impact on the anti-HIV activity of human NK (natural killer) cells (Arnold et al., 2013). Vincent Arnold, and co-authors suppose that an exogenous peptide S100A9 can be considered as the potential ligand for the control HIV-1 replication by NK cells (Arnold et al., 2013). Therefore, we can identify the existing novel approaches for HIV infection control that can be useful for the development of novel strategies to cure HIV. It also can help to create new hypotheses about potential mechanisms of HIV control leading to the development of new approaches to HIV treatment.

For some other proteins, there was no direct evidence of their role in HIV infection control and progression. But the analysis of their functions led to understanding that the differences in the expression of these proteins in the CD4+ or CD8+ T cells and some other immune cells may be associated with the velocity of HIV/AIDS progression. For instance, since HIV-1 glycoprotein 41 (gp) 41 prefers to interact with the cell-surfaced human leukocyte elastase (Bristow et al., 2003), one can suggest that the low levels of HLE expression can slow down the dissemination of HIV particles and therefore have an important role in HIV/AIDS progression.

There are experiments that provide the insights into CD8+ T cell response associated with the function of carcinoembryonic antigen-related cell adhesion molecule 1 (Khairnar et al., 2018). Base on the experiments carried out with lymphocytic choriomeningitis virus it was shown that carcinoembryonic antigen-related cell adhesion molecule 1 is essential for activation of CD8+ T cells. However, such results should be considered with awareness and more experiments are needed to adopt these hypotheses to HIV-1 viremic control.

Based on the text and data mining, we have earlier identified a set of proteins and discussed some molecular mechanisms shared by a novel coronavirus SARS-CoV-2 and HIV-1 (Tarasova et al., 2020). There were a few molecular pathways, including those related to immunology, autophagy, cell cycle regulation, shared by these two viruses if they infect humans. The present study is focused on the possibilities of text mining to extract data from the strictly relevant publications and investigate whether such an approach can address questions related to the aspect of biological studies. Our approach is based on a particular set of proteins that can be associated with the slow HIV/AIDS disease progression.

The two case studies aimed at the extraction of chemical names from the texts relevant to HIV reverse transcriptase inhibition, proteins and genes from the texts relevant to HIV control allow us to determine the advantages and disadvantages of text mining approaches to new information. The main advantage of text mining approaches is the possibility of covering the huge amount of textual data (Ruusmann and Maran, 2013; Capuzzi et al., 2017, 2018; Kandhro et al., 2017; Azam et al., 2019; Gambardella and di Bernardo, 2019; Guin et al., 2019; Ivanisenko et al., 2019; Alves et al., 2020). Text mining approaches allow retrieving the most recent and important information about chemicals, proteins, and genes associated with HIV treatment including their tissue-specific expression level (Ivanisenko et al., 2019). The main disadvantage is that our approach does not consider the expression level of the proteins extracted; this is due to the incomplete description of expression data in abstracts. On the other hand, we have evaluated the fully automated workflow for the purposes of extraction and analysis of the proteins or genes names that can be associated with the investigation of HIV ECs. Our study demonstrates that automated analysis of protein functions associated with HIV elite controllers allows us to hypothesize about the role of this protein in the HIV/AIDS progression. These observations and hypotheses may help to plan new experiments and develop new methods for HIV/AIDS treatment including the search for novel chemical compounds that can modulate the level of expression of target proteins, and vaccine development. We suppose that the suggested approach can be applied to an analysis of other viral infections, including those that have been affecting humanity for the last years (Basak et al., 2019; Tarasova et al., 2020; Tworowski et al., 2020). In addition it may help provide the possibility to analyze other various pathological processes non-related to viral infections that can involve the changes in gene expression (Kovalenko et al., 2016; Kandhro et al., 2017; Bizzarri et al., 2020) and find possible strategies to combat pathological conditions.

## Conclusions

In our study, we have developed and tested a new approach for automated extraction of named entities representing proteins and chemical compounds from the texts of scientific publications. Our method is based on the conditional random fields algorithm. We have developed a set of text features providing the reasonable accuracy of named entity recognition. We proposed the retrieval of named entities from the set of papers strictly relevant to (i) HIV reverse transcriptase inhibition and (ii) to the control of HIV/AIDS progression to test the ability of the algorithm developed to extract both the names of chemicals and proteins (genes). Our algorithm was tested on the retrieval of data on inhibitors of HIV reverse transcriptase. We were able to identify the HIV RT inhibitors with the precision 0.80 and recall 0.94. Then, we tested the applicability of our algorithm to identify a set of proteins potentially responsible for slow HIV/AIDS disease progression and HIV control. For this purpose, we collected a set of abstracts strictly relevant to the HIV elite controllers, a group of HIV positive patients who did not progress into HIV/AIDS for years in the absence of antiretroviral therapy. The extraction of proteins unique for the studies of HIV elite controllers allows us to identify the set of proteins responsible for the velocity of HIV/AIDS disease progression. Investigation of these proteins and their functions can provide insights into novel approaches for HIV/AIDS treatment.

## Data Availability Statement

The original contributions presented in the study are included in the article/Supplementary Materials, further inquiries can be directed to the corresponding author/s.

## Author Contributions

NB: provided realization, optimization and testing of the CRF algorithm, extraction of the names of chemical compounds and proteins/genes, computational experiments on the chemical data extraction, and manuscript preparation. OT: study design, preparation of test sets, collection of the abstracts of publication relevant to HIV reverse transcriptase inhibitors, HIV elite controllers, HIV-positive patients from NCBI PubMed and NCBI PMC databases, automated search of the extracted proteins/genes by their name in UniProt database, analysis of the results, manuscript preparation and review, and general supervision. SI: selection of the publications relevant to HIV elite controllers, manuscript review, and useful comments. VP: manuscript review, comments to the study design and to the manuscript content. All authors contributed to the article and approved the submitted version.

## Conflict of Interest

The authors declare that the research was conducted in the absence of any commercial or financial relationships that could be construed as a potential conflict of interest.

## Footnotes

^1^WHO HIV Incidence. Available online at: https://apps.who.int/gho/data/node.main.HIVINCIDENCE?lang=en (accessed October 15, 2020).

^2^Number of People (All Ages) Living With HIV. Available online at: https://apps.who.int/gho/data/view.main.22100WHO?lang=en (accessed October 15, 2020).

^3^BioCreative IV, CHEMDNER Corpus. Available online at: https://biocreative.bioinformatics.udel.edu/resources/biocreative-iv/chemdner-corpus/ (accessed August 21, 2020).

^4^BioCreative VI, ChemProt Corpus. Available online at: https://biocreative.bioinformatics.udel.edu/news/corpora/chemprot-corpus-biocreative-vi (accessed September 4, 2020).

^5^PubChem. Available online at: https://pubchem.ncbi.nlm.nih.gov/ (accessed November 20, 2020).

^6^UniProt. Available online at: https://www.uniprot.org/ (accessed November 20, 2020).
