# Supplementary material for: Automated Extraction of Information From Texts of Scientific Publications: Insights Into HIV Treatment Strategies
Source: Front Genet. 2020 Dec 22;11:618862. doi: 10.3389/fgene.2020.618862 (PMC7783389; doi:10.3389/fgene.2020.618862)
Supplement: Supplementary file 1 [file Data_Sheet_1.ZIP › Supplementary Material/Supplementary material.docx]

Supplementary Material


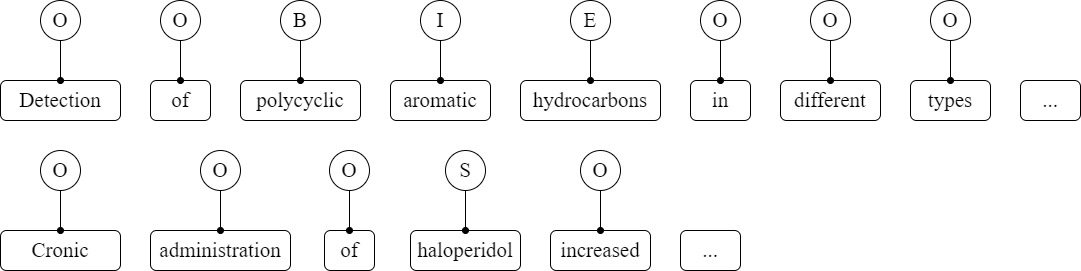


**Supplementary Figure 1**. Example of labeling tokenized text by SOBIE.


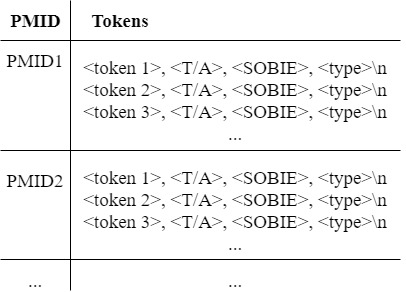


**Supplementary Figure 2.** Scheme of the database used for storing processed abstracts of publications.

**Supplementary Figure 3.** Precision, recall and F1-score comparison for CHEMDNER and merged CHEMDNER and ChemProt corpus for chemical named entity recognition.


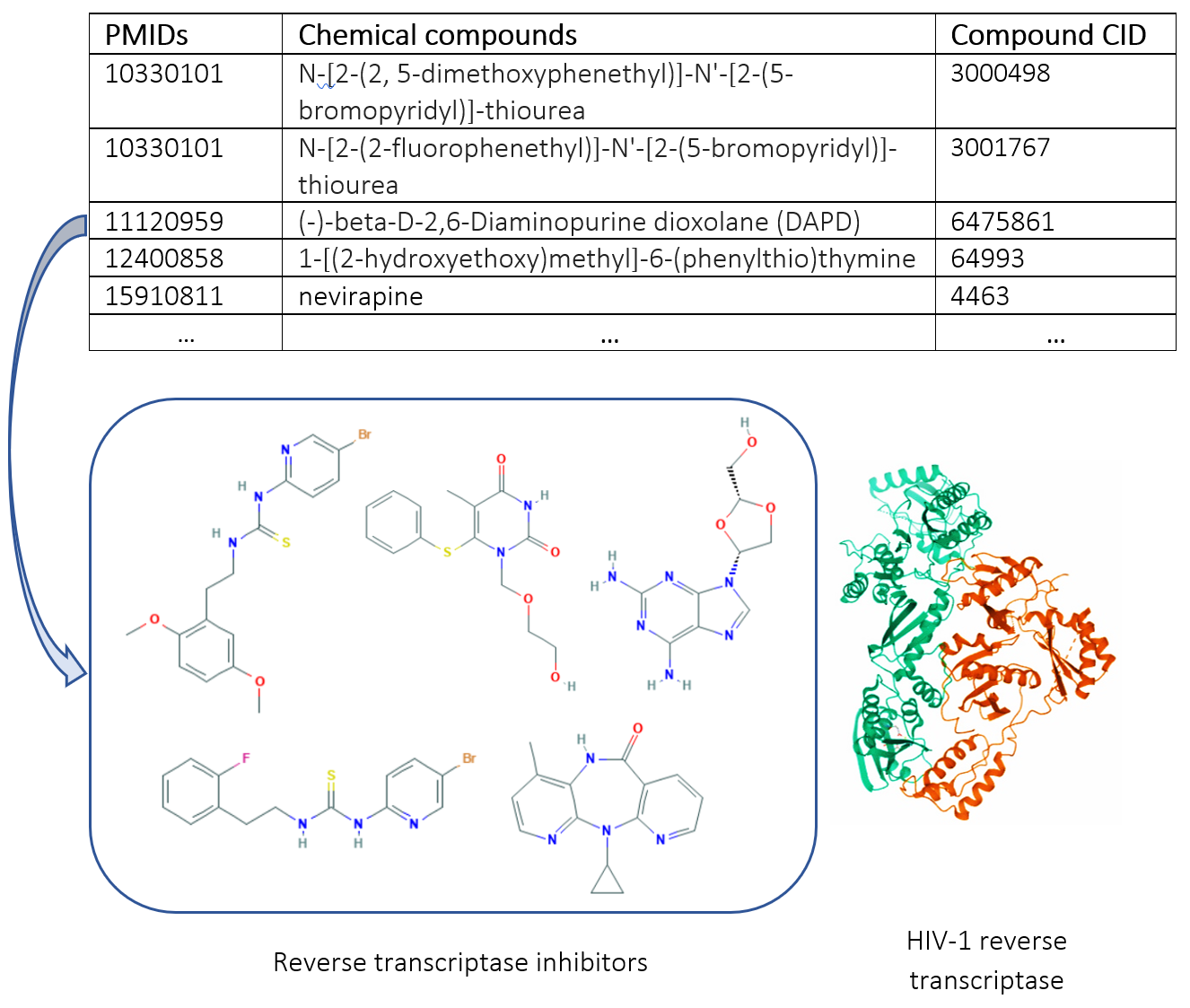


**Supplementary Figure 4**. Example of extracted inhibitors of HIV reverse transcriptase.

**Table S1.** Types of chemical compounds in CHEMDNER corpus.

| **Type** | **Example** |
| --- | --- |
| ABBREVIATION | ATP, HEPT |
| FAMILY | steroid hormones, ethers |
| FORMULA | C, S, C2H5OH |
| IDENTIFIERS | GRN-529 |
| MULTIPLE | nucleoside tri- and di-phosphates |
| SYSTEMATIC | sphingosine-1-phosphate |
| TRIVIAL | progesterone |

**Supplementary File 1.** Non-specific terms that were used for forming context features. (Non-specific terms.txt)

**Supplementary File 2.** Automatically extracted proteins from the texts dedicated to HIV-positive patients (Proteins HIV.txt)
